# Incorporating environmental heterogeneity and observation effort to predict host distribution and viral spillover from a bat reservoir

**DOI:** 10.1098/rspb.2023.1739

**Published:** 2023-11-22

**Authors:** Rita Ribeiro, Jason Matthiopoulos, Finn Lindgren, Carlos Tello, Carlos M. Zariquiey, William Valderrama, Tonie E. Rocke, Daniel G. Streicker

**Affiliations:** ^1^ School of Biodiversity, One Health and Veterinary Medicine, College of Medical, Veterinary and Life Sciences, University of Glasgow, University Avenue, Graham Kerr Building, Glasgow G12 8QQ, UK; ^2^ School of Mathematics, University of Edinburgh, Edinburgh, UK; ^3^ ILLARIY (Asociación para el Desarrollo y Conservación de los Recursos Naturales), Lima, Perú; ^4^ Yunkawasi, Lima, Perú; ^5^ Facultad de Medicina Veterinaria y Zootecnia, Universidad Peruana Cayetano Heredia, Lima, Perú; ^6^ National Wildlife Health Center, US Geological Survey, Madison, Wisconsin, USA; ^7^ Medical Research Council—University of Glasgow Centre for Virus Research, Glasgow, UK

**Keywords:** detection probability, Gaussian random field, partially observed species, species distribution models, vampire bat rabies

## Abstract

Predicting the spatial occurrence of wildlife is a major challenge for ecology and management. In Latin America, limited knowledge of the number and locations of vampire bat roosts precludes informed allocation of measures intended to prevent rabies spillover to humans and livestock. We inferred the spatial distribution of vampire bat roosts while accounting for observation effort and environmental effects by fitting a log Gaussian Cox process model to the locations of 563 roosts in three regions of Peru. Our model explained 45% of the variance in the observed roost distribution and identified environmental drivers of roost establishment. When correcting for uneven observation effort, our model estimated a total of 2340 roosts, indicating that undetected roosts (76%) exceed known roosts (24%) by threefold. Predicted hotspots of undetected roosts in rabies-free areas revealed high-risk areas for future viral incursions. Using the predicted roost distribution to inform a spatial model of rabies spillover to livestock identified areas with disproportionate underreporting and indicated a higher rabies burden than previously recognized. We provide a transferrable approach to infer the distribution of a mostly unobserved bat reservoir that can inform strategies to prevent the re-emergence of an important zoonosis.

## 1. Background

Mapping the geographical distribution of animal reservoirs of infection is a pre-requisite to anticipate and prevent spillover to other species [1]. For example, vaccination campaigns for canine rabies use detailed surveys of dog density to allocate vaccine distributions, and brucellosis control programmes require censusing of relevant hosts (cows, sheep and goats) to effectively implement testing, vaccination and slaughter [2,3]. However, for wildlife, distribution data are frequently sparse, presence-only records that do not cover the full geographical range [4,5]. The problem is exacerbated for species such as bats and rodents that are key reservoirs for zoonoses, but are difficult to observe given their small body size, reclusive nature, nocturnal activity or high mobility [4,6]. Species distribution models use relationships between field observations and environmental data to predict the occurrence or abundance of disease vectors or reservoirs [7]. More recent extensions of these models have been developed to deal with biases in input data and account for autocorrelation arising from geographical proximity between observations [4,8]. Accounting for such inferential challenges is vital to avoid misguided spatial allocations of interventions, but to date, such models have rarely been applied to wildlife disease reservoirs [5,9].

One of the most important zoonotic viruses transmitted by bats is rabies (genus *Lyssavirus*, family *Rhabdoviridae*), which causes an acute lethal encephalitis in all mammals [10]. In Latin America, the common vampire bat *Desmodus rotundus* (hereafter ‘vampire bat') is the main reservoir of rabies virus due to its population abundance, wide geographical distribution and obligate blood feeding, which provides a direct route for human and livestock infection via infectious saliva [11]. Efforts to mitigate the burden of vampire bat rabies (VBR) include human and livestock vaccination. However, financial and logistical challenges result in campaigns that are largely reactive to rabies outbreaks, and costs in human lives and livestock persist [12,13]. Rabies management also involves controlling vampire bat populations using topical anticoagulant poisons that spread between individual bats during social grooming or that are applied to cattle for later consumption by bats [14,15]. The ability of bat culling to reduce rabies incidence is controversial, and efficacy has been questioned based on field, phylogenetic and modelling studies [12,16–18]. Further, countries with active culling campaigns have seen spatial expansions of rabies virus into historically rabies free areas and growing disease burdens [19,20]. It is hypothesized that culls are ineffective because VBR is maintained by spatial processes, including wave-like invasions into historically rabies-free areas and metapopulation maintenance, but culls are reactive to rabies outbreaks and rarely synchronized across enzootic regions [17,19]. Improving the implementation of culls or the spatial distribution of human and livestock vaccines requires knowledge of the spatial distribution of vampire bat populations and how this distribution determines spillover risk. However, with rare exceptions, neither the location nor the number of vampire bat roosts are known with certainty [21].

Large scale models of the vampire bat distribution (e.g. across countries) have explored how climate and land use change might affect the future distribution of this species [22–24]. Predictions from these models are necessarily coarse given the wide variety of habitat types considered. By contrast, informing on-the-ground management requires accurate, high-resolution predictions of the density of both detected (i.e. observed roosts) and undetected roosts (i.e. roosts that remain undiscovered), necessitating more sophisticated models which account for spatial autocorrelation and explicitly incorporate heterogeneities in search effort. Here, we used data on the locations of vampire bat roosts (i.e. any place a wild vampire bat uses for shelter, excluding captures during foraging) in southern Peru where VBR is either endemic or invading historically uninfected zones [19] to: (1) identify the environmental and anthropogenic variables that drive vampire bat roost occurrence; (2) reconstruct the map of expected vampire bat roost density; (3) estimate the total number and spatial variation in undetected roosts; (4) test whether our predictions of vampire bat roost density improved explanations of the locations and intensity of rabies outbreaks in livestock; and (5) use the inferred projection of rabies outbreaks to re-assess the burden of rabies spillover.

## 2. Methods

### (a) Study area

**Figure 1.**
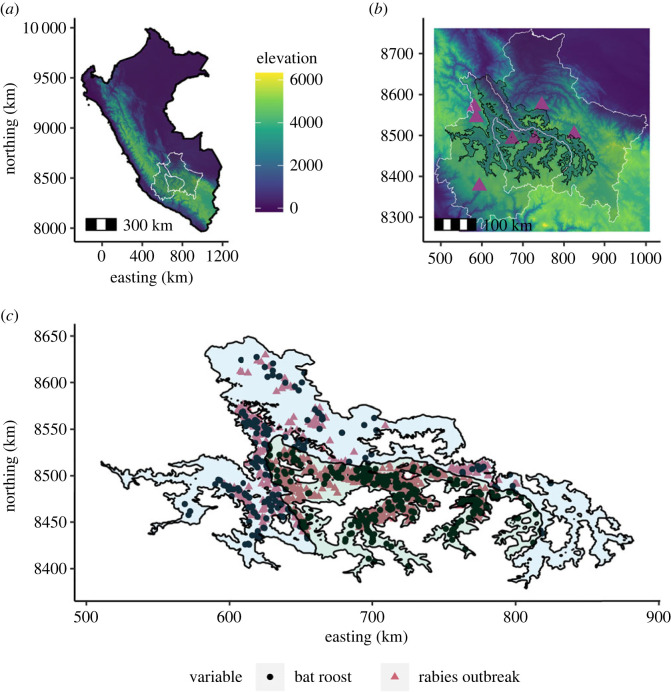
The study area with detailed spatial locations of vampire bat roosts and rabies outbreaks. (*a*) Elevation in Peru (source https://srtm.csi.cgiar.org) and the boundaries (white) of the departments (from left to right) of Ayacucho, Apurimac and Cusco. (*b*) Zoom to the three departments showing the study area, the inter-Andean valleys (amebae shape) and the SENASA offices (purple triangles). The valleys exclude landscape with elevation above 4000 m. (*c*) The study area with the two-level region factor (Ayacucho and Cusco in light blue, and Apurimac in light green), with the number of observed vampire bat roosts (*n* = 563) and the number of reported rabies outbreaks between 2003 and 2021 (*n* = 1212).

The study area comprised three neighbouring regions in southern Peru: Ayacucho, Apurimac and Cusco (joint area = 136 697 km^2^; figure 1). This area is composed of inter-Andean valleys, with elevations ranging from approximately 280 to 6000 m above sea level. To avoid modelling high-elevation areas that are known to exceed the physiological tolerance of vampire bats, we excluded areas above 4000 m (figure 1*b,c*) [19]. To improve the efficiency of downstream analyses, the smoothr package [25] in R (version 4.1.2 [26]) was used to simplify the boundaries the complex shapefile which resulted from excluding high-elevation areas.

### (b) Data on vampire bat roosts

We used the geolocations of 563 vampire bat roosts collected between 2007 and 2021 (figure 1*c*). Roosts included natural (68%) and human-made structures (32%). Most natural structures inhabited by vampire bats were caves (90%), with the remainder classified as trees (5%) or ‘other' (5%). Roosts in human-made structures were abandoned houses (47%), mines (24%), tunnels (15%) and ‘other' (14%). Data originated from three sources: internally from the research group (2.4% of observations), from the Regional Government of Apurimac (22%, derived from a 2-year rabies control campaign in 2014–2016, which included active searching for roosts in Apurimac) and from the National Service of Agrarian Health (SENASA, 75.6% of observations). For all three sources, roosts were geolocated by searching in areas with reports of vampire bat bites or livestock rabies outbreaks, typically with guidance from local communities. Hence, we jointly analysed unique roost locations from these three partially overlapping datasets. Coordinates of roost locations were projected in Universal Transverse Mercator (UTM zone 18S) and measured as northings and eastings in kilometres.

### (c) Variable selection

We considered a range of biologically plausible climatic, topographic and anthropogenic variables to explain the regional distribution of vampire bat roosts (electronic supplementary material, table S1 and figure S1). Climatic variables included minimum temperature of the coldest month and temperature and precipitation seasonality, which are known to influence the distribution of vampire bats at broad spatial scales [22–24]. We also assessed effects of annual mean temperature and annual precipitation to understand overall effects of temperature and precipitation on the local distribution of roosts and to estimate optimal climatic conditions. As landscape topography affects the distribution of vampire bats and these bats are thought to primarily disperse along rivers, we explored effects of elevation, slope and terrain ruggedness index [19,27]. We also included variables aimed to approximate the spatial distribution of structures that could act as vampire bat roosts (i.e. percentage of tree cover and its proxy, the above-ground biomass, and proportion of mines, number of rural population centres and human footprint). As vampire bats tend to roost in areas close to their food source and in proximity to water, we also included cattle density, percentage of crop cover (which is related to livestock presence) and the distance to the nearest river [13,27,28].

The variables we sought to model were available in different spatial resolutions. In general, we used the finest resolution available; however, we used bilinear interpolation to rescale certain variables to finer resolution when this was more appropriate for the hypothesized ecological effects. Specifically, we rescaled climatic variables from 1 km^2^ to 100 m^2^ under the assumption that microclimatic conditions at specific locations would influence their suitability as a roost. We also rescaled cattle density from 10 km^2^ to 5 km^2^ resolution based on knowledge of bat foraging distances [29,30]. Although this led to models in which covariates were considered at different spatial scales, sensitivity analyses found no substantive effects of rescaling on effect sizes or significance.

We assessed multicollinearity between variables by calculating variance inflation factors (VIFs). Since there is no established method to estimate VIFs in a Bayesian framework, we ran a negative binomial generalized linear model to calculate the VIFs among standardized variables. We adopted a threshold of 2.5 as an indicator of high levels of multicollinearity [31]. Variables with VIFs > 2.5 (namely, minimum temperature of the coldest month, continuous elevation, terrain rugosity index, above-ground biomass, annual mean temperature, precipitation seasonality and temperature seasonality) were excluded from the same model in Bayesian analyses (see below). In addition, since continuous elevation was highly correlated with the climatic variables, retaining continuous elevation in our model would have precluded including variables that are known to be important to explain vampire bats' distribution [22–24]. We therefore classified elevation to a binary layer indicating preferred and non-preferred environments for vampire bats based on the threshold of 3600 m of altitude [19].

### (d) Modelling approach

Vampire bat roost locations were treated as an inhomogeneous point process, a well-established framework for spatial modelling of presence-only data [32]. Specifically, roost density was modelled using a log Gaussian Cox process (LGCP), a process with intensity *λ*(*s*) at coordinates *s*. An LGCP is double-stochastic, as it is a hierarchical combination of a Poisson process at the first level (where the locations of the points are conditionally independent) and a Gaussian (or spatial) random field (GRF) at the second level to account for spatial correlation [33,34]. We selected an LGCP to take advantage of point locations to explain the intensity process that creates the observed roost distribution.

Gaussian random fields are spatially continuous structured random processes that typically have dense covariance matrices that are computationally demanding. An efficient way to introduce these structures in the model is to approximate the continuously indexed GRF by a spatially tessellated approximation to a stochastic partial differential equation [34,35]. For this, we used the Integrated nested Laplace approximation joint with a stochastic partial differential equation, implemented via the inlabru R package (development version 2.6.0.9003) [26,36,37]. We developed two main models that we present below: a detectability model to account for uneven observation effort in the study area, and the model of expected roost density, which was informed by the detectability model.

#### (i) Detectability model

To ensure the patterns identified by our model were not observation artefacts and to robustly estimate the total number of roosts, it was necessary to correct for the uneven observation effort. Because data on the number of person-hours spent searching for roosts was unknown, we used the spatial accessibility of each location to approximate search effort. We approached this as a pre-analysis step, akin to some distance sampling analyses that fit a detection function separately from the ensuing habitat modelling [38]. Specifically, the detectability model fitted roost detections as a flexible function of accessibility to the main source of roost detection, measured as the mean travel time (in minutes) separating each grid cell from any of the SENASA offices (figure 1*b*; electronic supplementary material, table S1 and figure S1) [39]. This was an appropriate metric to use because although SENASA is responsible for rabies and vampire bat control campaigns, and it is known that underreporting of VBR in livestock increases with the distance from SENASA offices, the location of SENASA offices is not related to vampire bat distribution [13]. When compared with Ayacucho and Cusco, Apurimac had the highest roost detection effort because searches were carried out by both national and regional governments, and our field studies in Apurimac started 7 years before than in the other two regions. Hence, to include the highest effort in Apurimac, we defined region as a two-level factor covariate separating Apurimac (highest effort) from Ayacucho and Cusco (combined) (figure 1*c*). The observation model was therefore defined by the expression

$$\lambda_{o}(s)=\exp(\beta_{0}+f(z)+\;\beta_{1}x_{\mathrm{Apurimac}}),$$

where *λ_o_*(*s*) is the detection probability at location *s*, *β*_0_ is the intercept term, *f*(*z*) is the nonlinear effect of the accessibility layer modelled using a random walk model of order 1 latent effect, and *β*_1_*x*_Apurimac_ represents the effect of region *x* (Apurimac is the reference). The predicted posterior mean derived from the observation model was normalized by assigning the probability 1 at the origin, which implies that a roost at the location of the SENASA offices is guaranteed to be known, resulting in the spatial probability of roost detection.

#### (ii) Matérn correlation model

The Matérn correlation model explains the scale of spatial dependency between neighbouring locations, capturing residual spatial patterns in roost density not explained by the available covariates. This correlation structure was expressed over a discretization of space known as the mesh (electronic supplementary material, figure S2), a finite grid of triangulations of the spatial domain that approximates smooth random effects within the model [40]. Here, the mesh was defined within the shapefile of the smoothed boundaries of the study area, and the resolution of the mesh was driven by the observed roost locations (hence directing more modelling detail in areas with detections). The correlation structure was defined according to the equation

$$\mathrm{cov}(i,j)=\sigma^{2}\mathrm{Matérn}(d_{ij},k),$$

where the covariance between any two locations depends on their distance (*d*), on the range (*k*) of the Matérn function and on the spatial variance (*σ*^2^). To obtain posterior distributions from the Matérn correlation model, this model included penalized complexity priors for the hyperparameters, range (*k*, practical range) and sigma (*σ*, the marginal standard deviation). These are non-informative, default priors available in inlabru which penalize model complexity [41].

#### (iii) Roost model

The expected density of vampire bat roosts (*λ*(*s*)) was fitted as a function of an intercept (*β*_0_), variables for roost distribution (*X*(*s*)), a spatial random field (*f*(*s*)) (formulated as a Matérn correlation model) and a spatial offset (*ε*). The offset is the probability of roost detection from the detectability model, to adjust for the uneven observation effort. The roost model is defined according to the following expression:

$$\lambda (s)=\exp\;(\beta_{0}+\beta X(s)+f(s)+\varepsilon ).$$

We began with a model with the offset and the spatial random field and added one variable at a time. Annual mean temperature and minimum temperature of the coldest month gave the lowest Watanabe-Akaike information criterion (WAIC) but were collinear, so we fitted two different models with these two variables. We used a forward addition procedure of variable selection based on WAIC to identify the most parsimonious model. For both models, we excluded variables that were collinear with temperature. We added the remaining variables (electronic supplementary material, table S1 and figure S1) one by one, choosing in each step the ones that provided best improvement in WAIC. We stopped when WAIC stopped improving.

Although intraspecific competition has not regularly been incorporated into models of bat distributions [4], this behaviour is sometimes observed in other colonial species (e.g. seabirds) [42]. We therefore explored competition between bat colonies by quantifying the pairwise Euclidean distances between roosts. As this analysis showed no evidence of repulsion among roosts which would be expected under a competition model, later models did not consider this possible effect (electronic supplementary material, figure S3).

#### (iv) Spatial predictions and estimates of abundance

An LGCP model predicts the intensity of a point pattern in continuous space at the resolution defined by the scale of spatial dependency between neighbouring locations. Predicting counts of roosts from the LGCP required defining a spatial grid to aggregate counts of roosts. For this purpose, the mesh was converted into a spatial polygon data frame. These conversions of space automatically resulted in a cell size of 8 km^2^ (i.e. 3.2 × 2.5 km). We then used the fitted LGCP model to predict the number of roosts in each grid cell, generating samples from the posterior of all model parameters. To integrate the effect of the spatial random field into roost counts, we projected integration weights to the mesh nodes and replaced the integral with a weighted sum in each grid cell. We predicted both the detected roosts (not correcting for the uneven effort) and the total expected roosts, in the latter case setting observation effort in all cells to the maximum such that observation probability was uniform. The posterior distribution of undetected roosts was calculated by subtracting the number of observed roosts (i.e. 563) from the density of the expected total count. We then estimated the posterior distribution for the total count that encompassed systematic stochasticity and uncertainty in parameter estimates, with and without adjusting for uneven effort.

#### (v) Model selection and validation

We performed model selection and validation by applying a spatial cross-validation method based on Valavi *et al*. [43], where blocks for aggregating the data and ensuring spatial independence were defined based on the effective range of spatial autocorrelation. The effective range of spatial autocorrelation was estimated using the posterior for the spatial random effect parameter in our model without covariates. This range (i.e. 30.53 km) was used to define regular subdivisions of space that could be assumed to be independent. We applied 10-fold cross-validation randomly, dividing the study area in blocks for training and testing (electronic supplementary material, figure S4). We computed the distance between predicted and observed number of roosts in the validation set as a pseudo-*R*^2^ for count data [44].

### (e) Explaining the distribution of past VBRV outbreaks

We hypothesized that our layer of predicted roosts would improve understanding of the distribution of past livestock rabies outbreaks (i.e. defined as a single spillover from vampire bats to livestock) relative to simpler representations of roost distribution, identify high-risk areas for future outbreaks, and identify locations with excess underreporting of outbreaks. For this purpose, we fitted a generalized linear model to the number of VBR outbreaks that occurred between 2003 and 2021 (1212 outbreaks; figure 1*c*; electronic supplementary material, figure S5). We used a negative binomial likelihood to account for the overdispersion in the counts of rabies outbreaks in livestock in each 8 km^2^ grid cell. The model included an offset describing the probability of reporting rabies outbreaks in each grid cell due to accessibility to the SENASA offices [13]. The same approach was used to estimate the offsets for the roost and rabies models. However, in the rabies reporting model, region was not included as fixed effect, as the three regions follow the national rabies surveillance programme. We compared the performance of four alternative representations of bat distribution: (1) the observed number of roosts in each grid cell (raw data); (2) a kernel smooth of the raw data, representing a naive interpolation of bat density based on observed data without covariates or bias correction; (3) the predictions of our LGCP model corrected for uneven observation effort; and (4) a smooth of these predictions (hereafter bat utilization distribution) using the same parameters as the smooth for raw roost data, as bat home range can be greater than 8 km^2^ [30,45]. In addition to one of the four representations of the vampire bat distribution, we included the number of months since the first rabies outbreak in livestock occurred in each district to account for the geographical expansion of VBR across the study area. We also included cattle density (electronic supplementary material, table S1 and figure S1), reasoning that rabies would be more detectable in areas with more cattle [11,19]. We compared the goodness of fit of eight models by ranking them according to the Akaike information criterion (AIC) and model deviance pseudo-*R*^2^. We used the best model to predict the spatial distribution of past rabies outbreaks with and without correcting for accessibility to reporting centres. To assess the burden of VBR spillover, we estimated the underreporting factor as the ratio between predicted rabies outbreaks (corrected by uneven accessibility) and the reported rabies outbreaks in each grid cell. This analysis was restricted to grid cells that had at least one reported rabies outbreak to avoid predicting outbreaks in areas that as of 2021, remained rabies free.

## 3. Results

### (a) Detectability and landscape correlates of roost establishment

**Figure 2.**
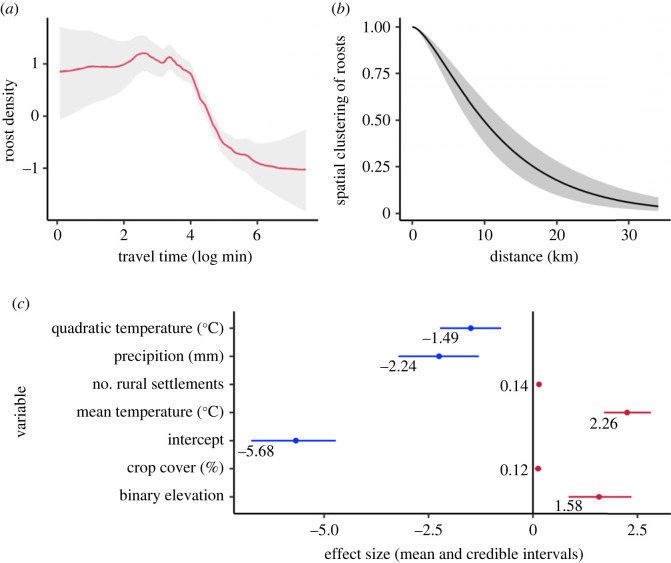
Factors affecting roost detectability and expected density: (*a*) Nonlinear effect of accessibility. The *x*-axis is travelling time (log minutes, i.e. exp (4) = 55 min, and exp (6) = 7 h) and the *y*-axis is the mode of roost density (in the linear predictor scale). (*b*) Range of the Matérn correlation model showing the mean correlation (*y*-axis) in function of the distance between points. The correlation range is on the log-intensity scale. Both in (*a*) and (*b*), the grey shaded areas show the 95% credible interval. (*c*) Posterior mean and respective 95% credible intervals of the covariates retained in the final model of roost density. Red for positive significant coefficients (i.e. positive credible intervals), and blue for negative significant coefficients (i.e. negative credible intervals). Binary elevation defines preferred (coded as 1) and non-preferred (coded as 0) habitats for vampire bats considering the threshold of 3600 m.

**Figure 3.**
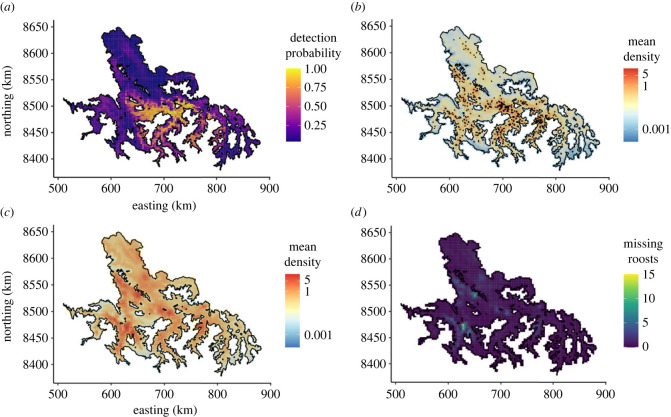
Spatial predictions of the detectability and roost models. (*a*) Probability of detecting one roost from the detectability model. The detection probability is a combination of accessibility and surveillance effort in each region. Yellow for high probability of roost observation and dark purple for low probability of roost detection. (*b*) Predictions of detected roosts. The black points are the 563 observed roosts. (*c*) Predictions of the expected roosts when adjusting for the uneven effort. For a better visualization of the spatial pattern of roost density, at the same scale, in both (*b*) and (*c*), the predicted posterior mean of roosts was mapped using the argument ‘trans' in log10. The maps in the linear scale can be seen in electronic supplementary material, figure S8. Both in (*b*) and (*c*) the same continuous colour gradient was applied with red indicating a higher mean of predicted roosts. (*d*) Predictions of hotspots of missing roosts (subtraction of maps *c*–*b*). Light colour indicates higher number of predicted missing roosts.

The detectability model showed that roost observation decayed in a sigmoid shape with increasing travel time to SENASA offices (figure 2*a*). The inflection point of the sigmoid indicated high roost detectability up to 55 min of travel, with detection probability approaching zero for roosts that were more than 7 h from any SENASA office. The probability of roost detection increased with accessibility and in areas that had more effort, particularly in Apurimac (figure 3*a*). The correlation structure of the Matérn model showed strong clustering of roosts up to distances of 10 km (half scale dependency) and positive correlations up to 30 km (figure 2*b*).

Vampire bat roost density was strongly influenced by climate, increasing with mean temperature until an optimum of 16.8°C (electronic supplementary material, figure S6) and decreasing in areas with higher average precipitation. Roost density was higher in areas with optimal elevation (less than 3600 m) and increased in proximity to rural human settlements and in areas with high crop cover (figure 2*c*).

### (b) Distribution and density of vampire bat roosts

The spatial projection of expected roost density resembled the observed roost distribution (pseudo-*R*^2^ = 0.60, figure 3*b*) and predicted a similar number of roosts as those used in model fitting (observed: 563 roosts; predicted: mean = 576, 95% credible interval [CI]: 528–625, electronic supplementary material, figure S7*a*). Pseudo-*R*^2^ decreased to 0.36 when removing the spatial random field, indicating that the GRF captured variability in roost density not explained by the environmental covariates. In our 10-fold cross-validation, the full model explained 45% of the variance in roost density. When correcting for uneven observation effort, our model estimated a total of 2340 roosts (CI: 2151–2541) (electronic supplementary material, figure S7*a*). As our model was trained using 563 known roosts, this result implies that approximately 1777 roosts (76% of 2340) remain undetected in our study area. The predictions of the posterior distribution for the total count of roosts had higher uncertainty when adjusting for effort, as it predicts the total abundance expected in the study area, accounting for observed and missing roosts (electronic supplementary material, figure S7*b*,*c*).

Importantly, roost detectability varied considerably over space, with as few as 4% of roosts known in some areas. Further, undetected roosts were spatially clustered, with hotspots identified in northern, western and southern areas (figure 3*c,d*).

### (c) Explaining the distribution of past VBRV outbreaks

**Figure 4.**
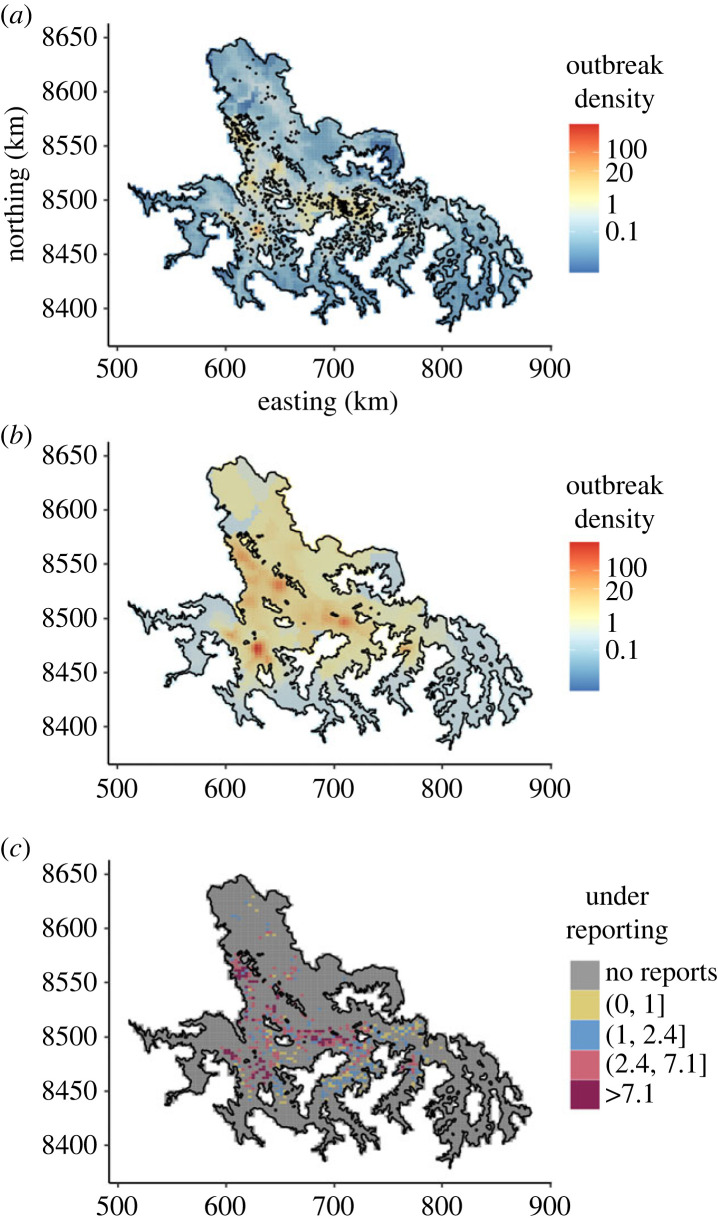
Spatial predictions of rabies outbreaks and heterogeneity in underreporting. (*a*) Predictions of reported rabies outbreaks. The black points are the 1212 reported rabies outbreaks in livestock between 2003 and 2021. (*b*) Predictions of rabies outbreaks after correcting for accessibility to reporting offices. Panels (*a*) and (*b*) have the same scale. A continuous colour gradient was applied, with red indicating a higher number of outbreaks. (*c*) Underreporting of rabies outbreaks in livestock. These values were calculated as the ratio between predictions corrected for accessibility (i.e. map *b*) and the 1212 reported outbreaks, hence only in cells with reported outbreaks (493 out of 4797 cells). The two thresholds used were the average (i.e. 7.1) and the median (i.e. 2.4) of underreporting.

In univariate models comparing how the alternative representations of the vampire bat distribution predicted rabies spillover, the bat utilization distribution derived from our roost predictions performed best, alone explaining 22% of the distribution of past rabies outbreaks (table 1). Adding covariates describing the geographical expansion of VBR across the study area and cattle density increased explanatory power by 15% (pseudo-*R*^2^ = 0.37, table 1). Models using the alternative representations of the bat distribution and the additional covariates had similar pseudo-*R*^2^ (0.31–0.36), but consistently higher AIC (*Δ*AIC = 131.1–25.2, table 1), hence were worse models. Spatial predictions of rabies outbreaks from the best model, generated with and without correcting for accessibility to reporting offices, revealed strong spatial heterogeneity, signalling hotspots of rabies outbreaks in livestock in western and northern areas (figure 4*a,b*). Comparing predicted to observed rabies outbreaks showed that, on average, the number of rabies cases in livestock was 7.1 times (95% CI: 3.9–10.4) higher than officially reported (figure 4*c*).

**Table 1.** Estimate and 95% confidence intervals of the covariates included in the eight models used to compare the performance of four representations of bat distribution. The estimates of the fixed effects are presented in the logarithm and standardized forms. Models are displayed from lower to higher AIC, and higher to lower pseudo-*R*^2^, both identifying model 1 as best model. Note: The pseudo-*R*^2^ of a multivariable model only with cattle density and the number of months since first rabies outbreaks, is 0.28.

model	covariates							AIC	pseudo-*R*^2^
	bat distribution				cattle density	interaction cattle and bat distribution	months since first rabies outbreak		
	roost data	smooth roost data	roost model	bat utilization distribution					
1				0.53 [0.46; 0.61]	0.12 [0.03; 0.22]	0.17 [0.07; 0.27]	1.08 [0.96; 1.19]	3798.1	0.37
2			0.53 [0.46; 0.60]		0.13 [0.04; 0.23]	0.13 [0.04; 0.22]	1.13 [1.02; 1.25]	3823.3	0.36
3		0.54 [0.46; 0.62]			0.07 [−0.04; 0.17]	−0.07 [−0.17; 0.04]	1.12 [1.0; 1.24]	3842.6	0.35
4	0.31 [0.23; 0.38]				0.18 [0.08; 0.28]	−0.004 [−0.10; 0.09]	1.26 [1.15; 1.38]	3929.2	0.31
5				0.88 [0.81; 0.96]				4105.0	0.22
6			0.90 [0.83; 0.97]					4159.6	0.19
7		0.80 [0.73; 0.88]						4175.3	0.18
8	0.41 [0.33; 0.49]							4368.0	0.06

## 4. Discussion

Focusing on an important bat reservoir in Latin America, our study provides a transferrable statistical approach to infer the spatial distribution of partially observed wildlife. In our study, spatial heterogeneity in vampire bat colonies was associated with topographic, climatic and anthropogenic factors but also strongly affected by previously unrecognized observation biases. Indeed, our results suggest that only 24% of vampire bat roosts are known to authorities, and models correcting these biases revealed previously undetected hotspots of vampire bat roosts in the northern, southern and western areas. Finally, predictions of vampire bat roost density improved models of the locations and intensity of historical rabies outbreaks, revealed hotspots of rabies outbreaks in livestock in the northern and western areas, and increased the projected burden of rabies mortality. Our results might improve the allocation of resources for VBR prevention by identifying previously unrecognized areas at high risk for viral incursion (i.e. areas with hotspots of undetected vampire bat roosts that are still rabies free) and endemically infected areas that are hidden to surveillance systems.

Our results show that temperature and precipitation influence not only the large-scale geographical range of vampire bats, but also the local intensity of roosts [22–24]*.* Although the specific mechanism by which precipitation acts on vampire bat distribution is poorly understood [30], the negative effect we observed reflects the increase in observed roosts from the rainy north to the dry climate in the centre and south of our study area [46]. Temperature effects were expected as vampire bats don't survive in areas with environmental temperatures below 10°C or above 37°C [14,29,47]. Of note, however, the optimal environmental mean temperature found in our study (i.e. 16.8°C) is relatively low compared with the recommended temperature for maintaining *D. rotundus* in captivity, between 21°C and 27°C [48]. It is possible that this difference reflects our use of ambient temperature rather than within roost temperature, which could be a better proxy of the recommended temperature in captive conditions. Although relationships between within roost and ambient temperature may vary depending on local conditions and roosts characteristics, temperatures within roosts would generally be expected be lower, not higher than ambient temperatures, particularly in daytime hours when bats occupy roosts [49]. Our results indicate that vampire bats in our study area exist in sub-optimal temperatures. Indeed, the inter-Andean valleys we studied represent a range limit for this species in southern Peru, with higher elevation areas farther south experiencing intolerable conditions. This implies the possibility of physiological trade-offs that might influence the susceptibility to or tolerance of vampire bats to infection [17]. Effects of marginal habitats on infection and immunity have been previously reported in several taxa. For example, female tree swallows (*Tachycineta bicolour*) in suboptimal sites in Alaska had impaired immunity, and populations of whistling tree frogs (*Litoria verreauxii verreauxii*) in low-quality habitats were less likely to persist through epizootics of chytridiomycosis [50,51]. For vampire bats, heightened susceptibility to lethal rabies infection is predicted to have large dynamical consequences on viral prevalence and spillover [17]. If impaired immunity in these populations is verified, we speculate this might contribute to the disproportionate burden of rabies in our study area, which despite its small size, accounts for most rabies outbreaks at the national level [19]. It is also conceivable that the high genetic divergence of vampire bats in our study area from those in tropical areas included physiological adaptations to low temperatures which may have unpredictable trade-offs with immunity [52–54]. Potential effects of marginal habitats on vampire bat immunity is a subject worth addressing in future studies.

We also found effects that are consistent with the influence of human activities on vampire bat roost establishment. The positive effects of the number of rural human settlements and crop cover on vampire bat roost establishment likely reflect the availability of livestock, which are the main food source for *D. rotundus* [28,55,56]. Although it is unexpected that livestock itself was not retained by the model, we suggest that the global layers used, while suitable to access broad scale effects of livestock, are less representative of variation in livestock density at fine spatial scales than the more finely resolved measures of human presence [56]. As such, our findings support the hypothesis that anthropogenic activities favour vampire bat roost establishment and may facilitate spatial expansions of this species to new areas within climatically tolerable regions [52].

Despite the influence of environmental variables in explaining vampire bat roost density, the higher pseudo-*R*^2^ after adding the GRF (pseudo-*R*^2^ = 0.36 versus 0.60) indicated that the spatial dependency among roosts explained considerable variation in roost density. In addition, the spatial clustering of vampire bat roosts decayed with the distance (figure 2*b*). This result suggests the absence of competition between vampire bat roosts, which would have been expected to generate a negative correlation at small distances [33]. In other taxa such as cranes and seabirds, spatial dependency arises from dispersal limitations, interspecific competition, disturbance, or social interactions [8,9,33]. Here, social interactions may be particularly important as vampire bats are a social species and often use multiple roosts mostly within small areas (2 to 3 km radius) [57,58]. Given that vampire bat home ranges are believed to extend only 5–10 km, it was unexpected that the correlation between roosts in our study area remained positive up to 30 km (figure 2*b*) [29,30]. It is conceivable that longer distance connectivity—occasionally reported—may be more common than previously recognized [28,45]. Regardless, both clustering and long-distance movement have important implications for rabies spatial spread which are not currently captured in existing epidemiological models [21]. More generally, our results illustrate the value of accessing the shape of the Matérn correlation range to understand and generate hypotheses about animal ecology and social behaviour.

Observation biases are likely to be widespread in datasets of reclusive species [5]. Our observation model captured a decay in vampire bat roost detection as accessibility to reporting offices decreased (figure 2*a*) and a sharp decrease in observed roosts in Ayacucho and Cusco (figure 3*a*). When correcting predictions of the roost model for the uneven effort, our model estimated that the number of undetected roosts was more than triple the number of known roosts. Moreover, undetected roosts were spatially clustered, rather than uniformly spread across the study area. Of note, some hotspots of undetected roosts are in currently rabies free areas that have neighbouring areas with active viral circulation, indicating risks of rabies incursions into high risk areas that would be primed for large outbreaks in livestock [19]. This highlights the importance of incorporating observation processes into model projections, enabling interventions such as preventive bat culls or livestock vaccination that might prevent spillover or reduce its burden in high-risk areas. More generally, improved understanding of the spatial distribution of vampire bats facilitates spatially synchronized control, which is predicted to be central to successfully managing rabies in endemic areas, but until now, was precluded by gaps in our knowledge of bat distribution [17,27,56].

Our predictions of detected and undetected roosts improved understanding of the spatial distribution of VBRV outbreaks in livestock and revealed hotspots of disease underreporting. Previous work demonstrated a positive correlation between detected bat roosts and density of rabies outbreaks in Brazilian livestock [27,28]. We found that the modelled roost distribution performed better than the raw data of roost detections for explaining the spatial pattern of past rabies outbreaks (table 1). This suggests that more refined representations of the distribution of vampire bats may improve models of rabies spread, although we acknowledge that differences between the performance (i.e. pseudo-*R*^2^) of models with the smoothed raw data and roost predictions were relatively small. This may reflect the fact that roost detection and notification of rabies outbreaks were generated by similar observation processes, both being affected by distance from the nearest reporting centre. Nevertheless, the ability to account for undetected bat roosts is novel and advantageous for identifying high risk areas for spillover, which can be targeted to strengthen rabies awareness (i.e. increasing chances of reporting), surveillance and control (i.e. vaccination and synchronized culling, bat vaccination) [59].

In Latin America, rabies is considered a neglected zoonosis as defined by the World Health Organization, and suspected outbreaks in livestock are notified via passive surveillance [60]. Hence, underreporting of rabies outbreaks leads to underestimation of the true burden of the disease [10,13,55]. In previous research, Benavides *et al*. [13] used questionnaires to estimate underreporting in the inter-Andean valleys of Ayacucho, Apurimac and Cusco, predicting that mortality from VBRV was 4.6 times (95% CI: 4.4–8.2) higher than officially reported, and inferring spatial heterogeneity in underreporting at the district level. Here, without the use of questionnaires, our model estimated that between 2003 and 2021, mortality from VBRV in the study area was 7.1 times (95% CI: 3.9–10.4) higher than officially reported. In addition, our predictions of rabies underreporting demonstrated spatial heterogeneity at a finer resolution (i.e. 8 km^2^ instead of at district level) than previously possible (figure 4*c*). These results have two distinct implications. First, the elevated estimate of underreporting implies an underestimation of the true burden of rabies which can be used to inform VBR management. Second, our ability to estimate underreporting at fine spatial scales empowers more precise geographical allocation of educational campaigns to encourage reporting where they are most needed.

Although the statistical approach presented here provides advances in modelling partially observed wildlife-disease reservoirs, our projections may be improved in several ways. Future work could explore the unexplained variation of our model, such as missing covariates, different relationships between covariates and the response, and incorporate the temporal scale of the data. Although a major contribution of this work was to use modelled detection probabilities to correct for observation biases implicit in presence-only data, future modelling could incorporate absence (i.e. locations where roosts were not found) or effort data (e.g. number of person-hours surveying) [4]. Future work could also take advantage of more sophisticated extensions of species distribution models that integrate distinct but complementary data on vampire bat occurrence (e.g. roosts and reports of bat bites) [33]. In addition, integrating the observation and the roost models, rather than modelling them separately, would be advantageous to fully propagate uncertainty across models [61,62]. Finally, predicting rabies outbreaks in livestock requires not only information on local conditions, such as roost distribution and livestock density, but also needs to consider the spatio-temporally dynamic nature of rabies [19].

In conclusion, we present a transferrable statistical approach to model the spatial distribution of difficult to observe species. To our knowledge, this is the first-time observation effort and spatial autocorrelation have been used to reconstruct the number of likely roosts for any bat species. Correcting density predictions for uneven effort provided qualitative and quantitative gains, identifying putative hot and cold spots of vampire bat roosts, high-risk areas for VBRV incursions, and highly resolved areas of disproportionate underreporting of rabies outbreaks. These results can be valuable in spatial models that explore determinants of viral maintenance and spillover risk and might guide effective monitoring of *D. rotundus* and prevention and control strategies for VBR. More generally, our results show how incorporating existing and routinely collected data into robust statistical models can improve the management of zoonoses.

## Data Availability

The data and R scripts that support the findings of this study are available in Zenodo (https://doi.org/10.5281/zenodo.10026623) [63]. Supplementary material is available online [64].
